# An eight-year follow-up study of Home Invasive Mechanical Ventilation in Finland

**DOI:** 10.1186/s12890-024-03263-8

**Published:** 2024-09-19

**Authors:** Hanna-Riikka Kreivi, Petra Kotanen, Waltteri Siirala

**Affiliations:** 1 HUH Heart and Lung Center, Helsinki University Hospital and University of Helsinki, Haartmaninkatu 4, P.O. Box 372, Helsinki, FI-00029 Finland; 2 Department of Anesthesiology and Intensive Care, Turku University Hospital and University of Turku, Turku, Finland

**Keywords:** Home mechanical ventilation, Home invasive mechanical ventilation, Chronic respiratory failure, Non-invasive ventilation

## Abstract

**Background:**

Studies on long-term invasive mechanical ventilation (IMV) *via* tracheostomy in chronic respiratory insufficiency are limited. The aim of this study was to clarify the use of HIMV (home invasive mechanical ventilation) within the Finnish population and to analyze the characteristics and survival rate of HIMV patients from 2015 to 2022.

**Methods:**

Data on HIMV patients was collected annually from all Finnish Hospital District patient registries between January 1, 2015, and December 31, 2022. Data included basic demographic data of the patients, underlying diagnosis, time from diagnosis to HIMV initiation, treatment duration, and mortality.

**Results:**

This study included 179 patients. In 2015, there were 107 HIMV patients, and as of December 31, 2022, there were 95 patients. During the eight-year follow-up period, 84 patients (46.9%) died and there were 67 new patients between 2015 and2022. The prevalence of HIMV treatment in Finland was 2.4/100,000 on January 1,2015, and 1.8/ 100 000 on December 31, 2022. The average number of years living with HIMV for deceased patients at death was 10.1 ± 10.5 years largely depending on the underlying diagnosis. Of all the HIMV treatments, 32% were elective.

**Conclusions:**

HIMV is a rare treatment in Finland, and based on our 8-year follow-up, prevalence of HIMV is diminishing. Given the high demands, and significant costs associated with HIMV, it is essential to prepare for long treatment, when planning HIMV. It is also advisable to prolong non-invasive ventilation (NIV) treatments for as long as possible.

## Introduction

Home mechanical ventilation (HMV) serves as a long-term treatment for patients with chronic respiratory insufficiency. HMV can be delivered non-invasively via an interface (non-invasive ventilation, NIV) or invasively, at home, involving an artificial airway inserted directly to the trachea (home invasive mechanical ventilation, HIMV). NIV is a simple method and has become increasingly common for assisted ventilation, so much so that patients can often manage this on their own. In a recent study by Toussaint et al. [1] a minimal budget for NIV equipment was estimated at approximately €168/patient/year and with services; with maintenance included, the budget could increase to between €3232 and €5760/patient/year. In contrast, HIMV represents a method of life support ventilation that demands advanced expertise and 24-h caregiving, thereby resulting in annual costs ranging from €400,000 to €500,000 per year in Finland. Comparing the cost of HIMV in different countries is challenging due to significant variations in health care systems, national medical fees, health insurances, and wages for caregivers [2]. However, it is evident that HIMV is a remarkably more expensive treatment modality than NIV.

In Finland, legislation guarantees equal rights for HIMV-dependent patients regardless of the socioeconomic status of patients or underlying diagnosis [3]. In 2015, there were 107 HIMV patients in Finland and during the 4-year follow-up, there were 34 new patients (24.1%) and 46 patients (32.6%) had died [4]. The prevalence of HIMV in Finland was 2.4/100,000 in2015 and 2.0/100,000 in 2019 [4]. The mean duration of HIMV among deceased patients was 11 years [4]. Nevertheless, our earlier study has been questioned for its short follow-up period. Despite our efforts and the substantial annual costs associated with HIMV, our healthcare system still lacks a public registry that documents the prevalence of HMV patients and long-term effects of both NIV and HIMV on life expectancy.

Given the limited prior research on long-term HIMV treatment as well as its effect on life expectancy and quality of life (QoL), we found it important to continue our study on Finnish HIMV patients. The aim of this study was to clarify the use of HIMV within the Finnish population and to examine the characteristics and survival of HIMV patients from 2015 to 2022.

## Methods

This study was a longitudinal cohort study. We collected data on Finnish HIMV patients yearly, every year on January 1, 2015–2022 and then on December 31,2022. We received permission from Finnish Institute for Health and Welfare (THL/769/5.05.00/2017) to collect information on all HIMV patients in Finland. According to Finnish legislation, patients’ consent is not required to survey studies in Finland. The Medical Ethics Committee of the Hospital District of Southwest Finland approved the study protocol (study number 97/1802/2014).

We contacted all twenty hospitals in all five university hospital regions in Finland that treat adult HIMV patients (≥ 16 years) personally by telephone and by email. The physicians responsible for HIMV patients were asked to fill in a questionnaire providing information on the diagnosis underlying chronic respiratory failure that leads to HIMV, time from diagnosis to HIMV initiation, duration of HIMV treatment, mortality, and weaning during eight-year follow-up period. All patients who required invasive mechanical ventilation (IMV) delivered via a tracheostomy for over three months were included in the study. Information on Finland’s population was taken from Statistics Finland, a Finnish governmental authority. The population of Finns aged 16 years and over was 4.5 million at the end of the year 2014 and 4.7 million at the end of the year 2022.

Patients were divided into seven diagnostic groups according to their underlying disease: amyotrophic lateral sclerosis (ALS), Duchenne muscular dystrophy (DMD), post-polio syndrome, spinal cord injuries (SCI)(including spinal cord diseases and injuries), other muscle dystrophies (MD) (e.g., dystrophica myotonica, limb-girdle muscular dystrophy, nemaline myopathy, and Becker’s dystrophy), other neuromuscular diseases (e.g., polymyositis, spinal muscular atrophy, Guillain-Barré syndrome, and other polyneuropathies), and miscellaneous (e.g., congenital deformities, congenital or secondary central sleep apnea, and conditions due to brain tumors or injuries, intracerebral infarctions, or hemorrhages, and encephalitis).

### Statistical analysis

Statistical analysis was conducted using SPSS, version 25 (IBM SPSS Statistics). The results are presented as N ± SD or mean values ± SD. An independent sample t-test, Chi-square test, and Kruskal Wallis test were used to analyze significant differences between the groups, and a p-value < 0.05 was considered statistically significant.

## Results

We received responses to study questionnaires from all hospital regions in Finland. The characteristics of the patients are described in Table 1. Overall, there were 179 patients in the study, of which 95 were alive as of December 31, 2022. During the entire eight-year follow-up period, of all patients, 84 patients (46.9%) died. More than half the patients with post-polio syndrome, ALS, and DMD died (Table 1). Altogether, 82.8% of the patients were dependent on HIMV, and the mean usage was 22.3 ± 4.5 h. DMD patients had the highest HIMV usage at 24 h, while the miscellaneous group had lowest usage 20.1 ± 5.4 h. There were 112 patients whose HIMV treatment began before January 1, 2015, and between 2015 and 2022 there were 67 new patients. Survival at the end of the follow-up period for the patients who began HIMV treatment before and after 2015 was 44.6% (50 patients alive) and 67.2% (45 patients alive), respectively. The prevalence of HIMV treatment in Finland in the population aged 16 and over was 2.4/100.000 as on January 1,2015, and 1.8/100.000 on December 31,2022.

**Fig. 1:**
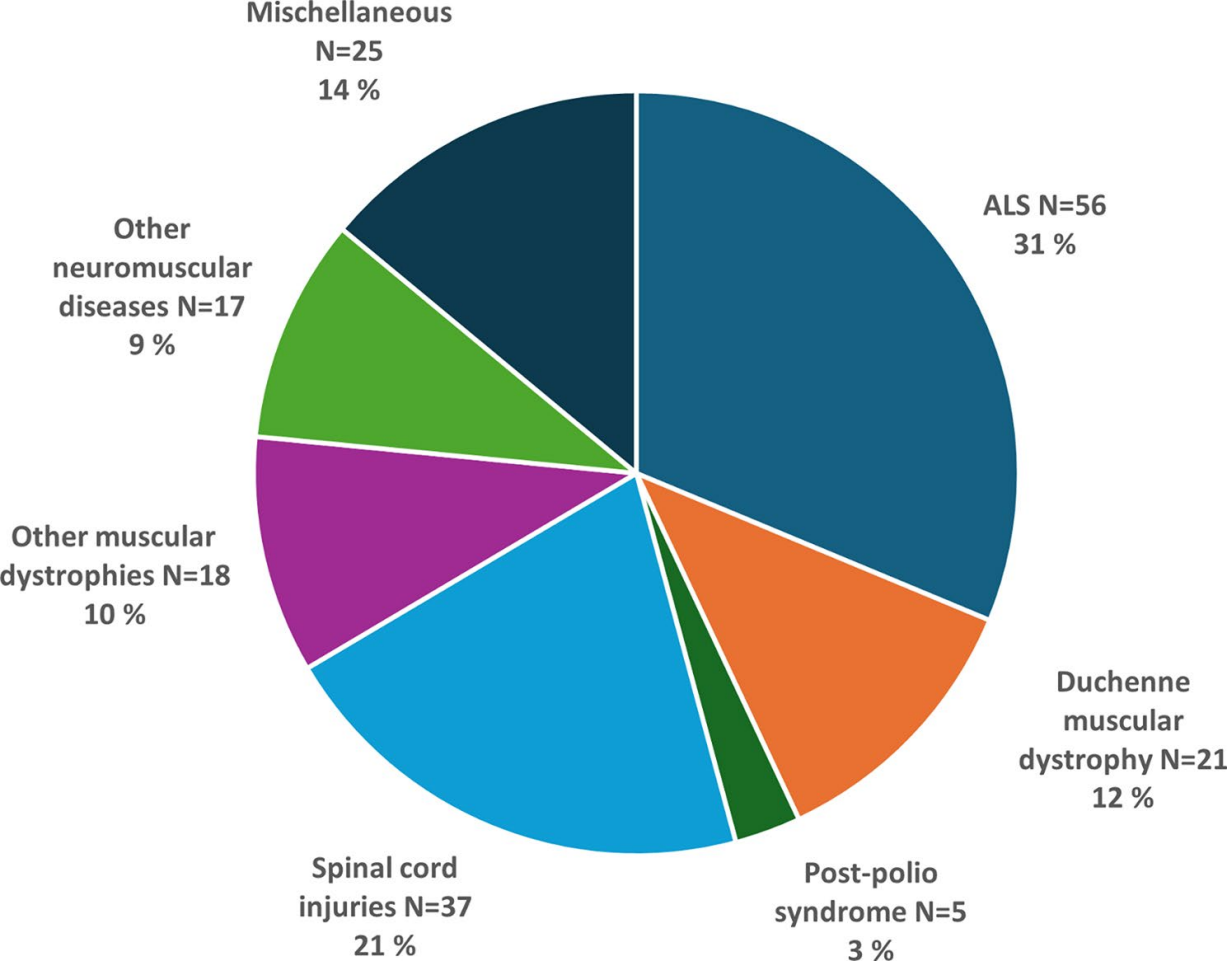
Number and percentage of patients receiving home invasive mechanical ventilation divided by diagnosis on December 31, 2022

**Fig. 2:**
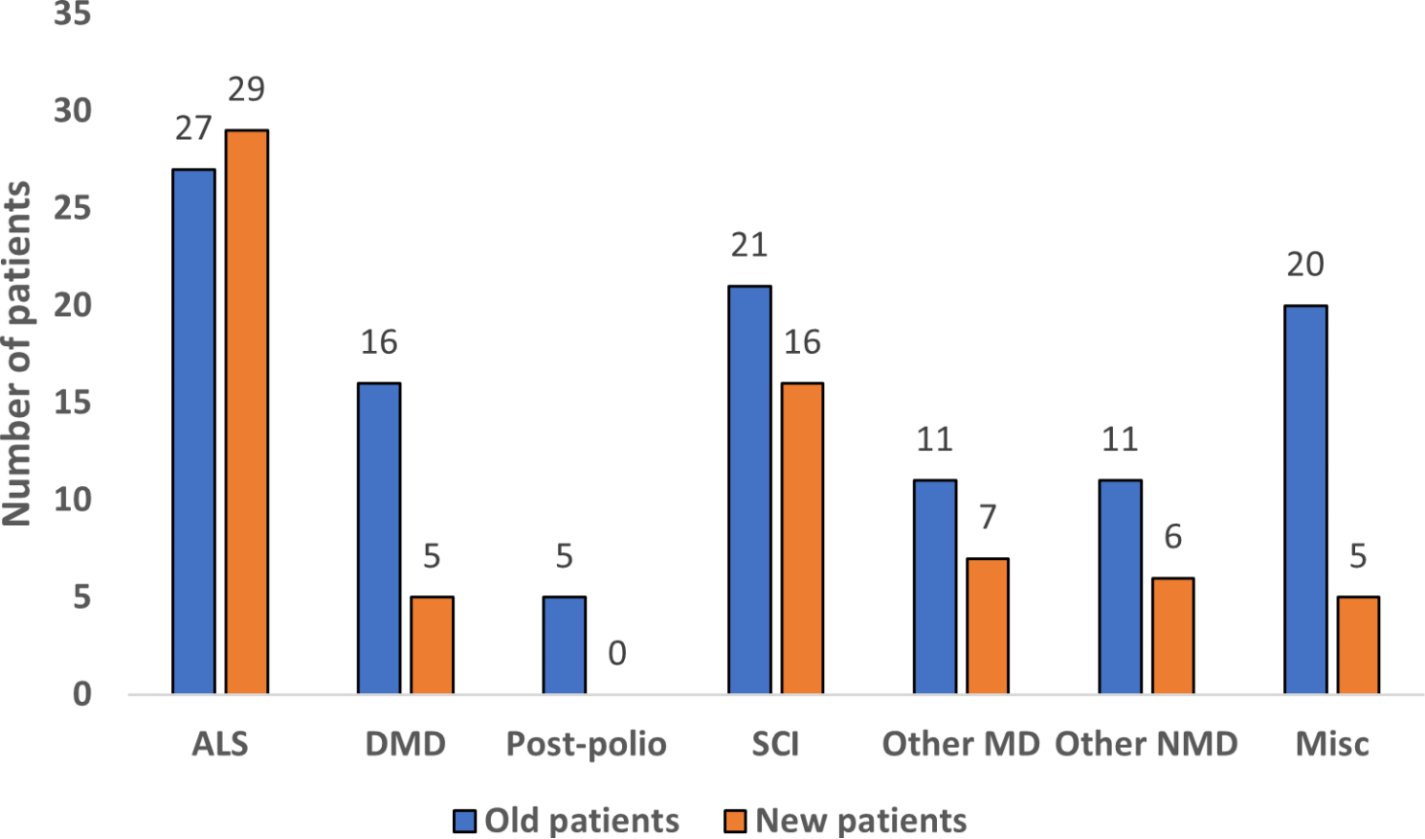
Number of the old (treatment initiated before 1.1.2015) and new patients (treatment initiated after 1.1.2015). Abbreviations: ALS= amyotrophic lateral sclerosis, DMD= Duchenne muscular dystrophy, SCI= spinal cord diseases and injuries, MD=muscular dystrophies, NMD= neuromuscular diseases, Misc= miscellaneous

The diagnostic groups of the patients are presented in Fig. 1. The most common underlying conditions leading to HIMV were ALS, SCI, and muscular dystrophy. When comparing the patients whose treatment was initiated before and after January 1, 2015, most of the new patients suffered from ALS and SCI. (Fig. 2). The mean age of patients whose HIMV treatment began before 2015 was 43.1 years and that of those whose HIMV treatment began after January 1, 2015, was 50 years.

**Fig. 3:**
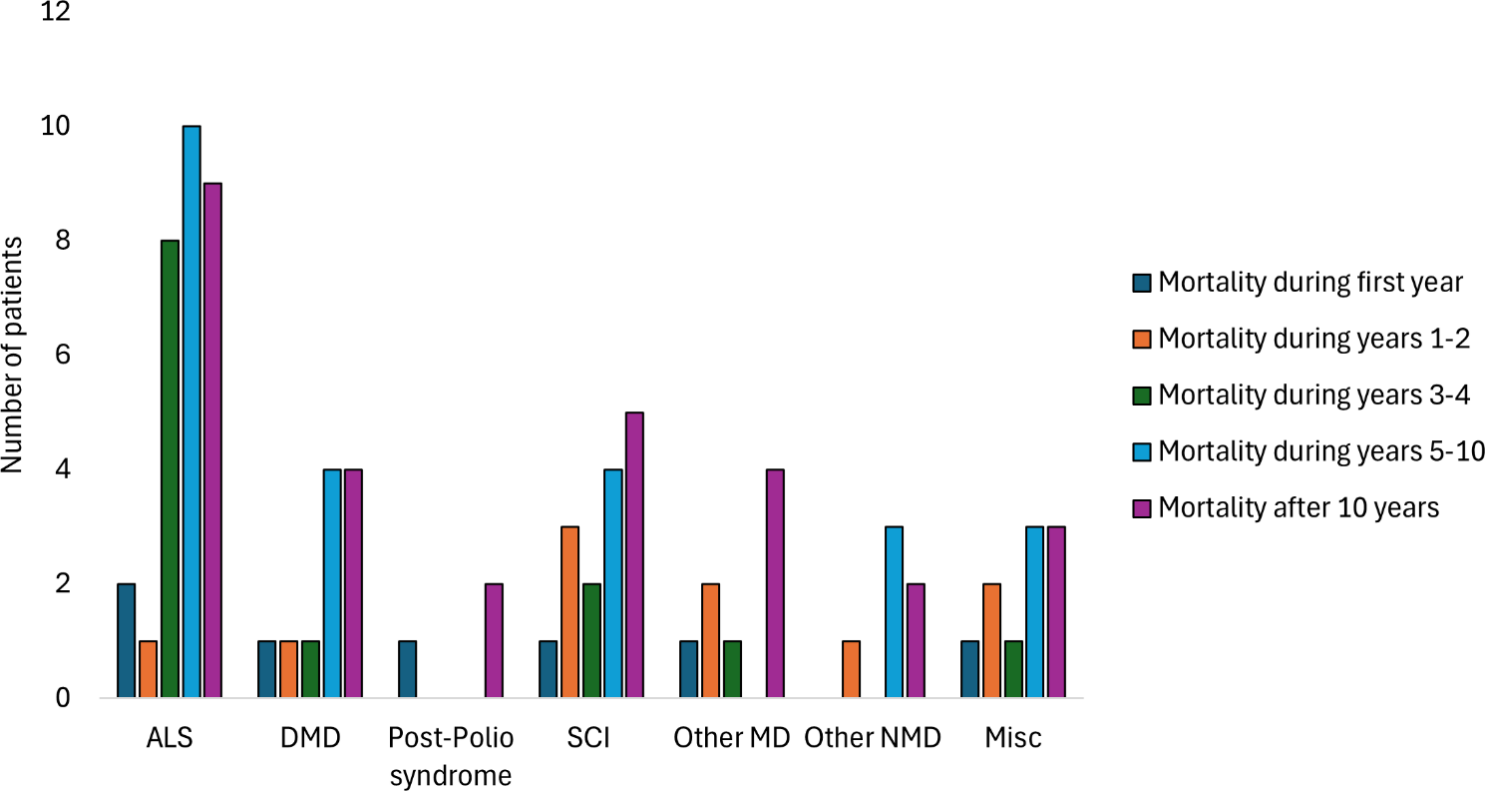
Mortality of the patients (number of patients) during follow-up 1.1.2015–31.12.2022 divided in groups by number of HIMV treatment years. Abbreviations: ALS= amyotrophic lateral sclerosis, DMD= Duchenne muscular dystrophy, SCI= spinal cord diseases and injuries, MD=muscular dystrophies, NMD= neuromuscular diseases, Misc= miscellaneous

**Fig. 4:**
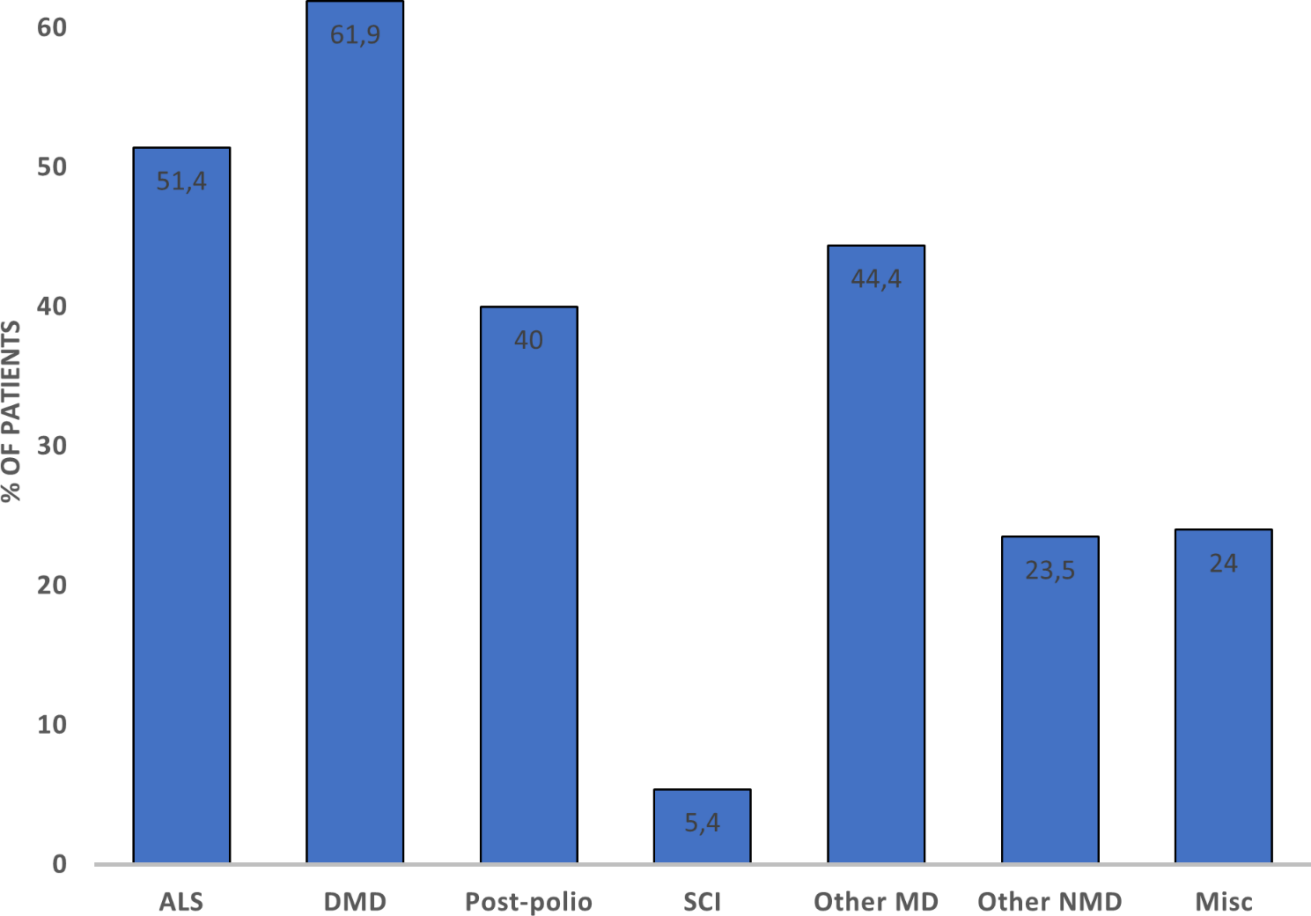
Electively initiated (%) home invasive mechanical ventilation treatments. Abbreviations: ALS= amyotrophic lateral sclerosis, DMD= Duchenne muscular dystrophy, SCI= spinal cord diseases and injuries, MD=muscular dystrophies, NMD= neuromuscular diseases, Misc= miscellaneous

**Fig. 5:**
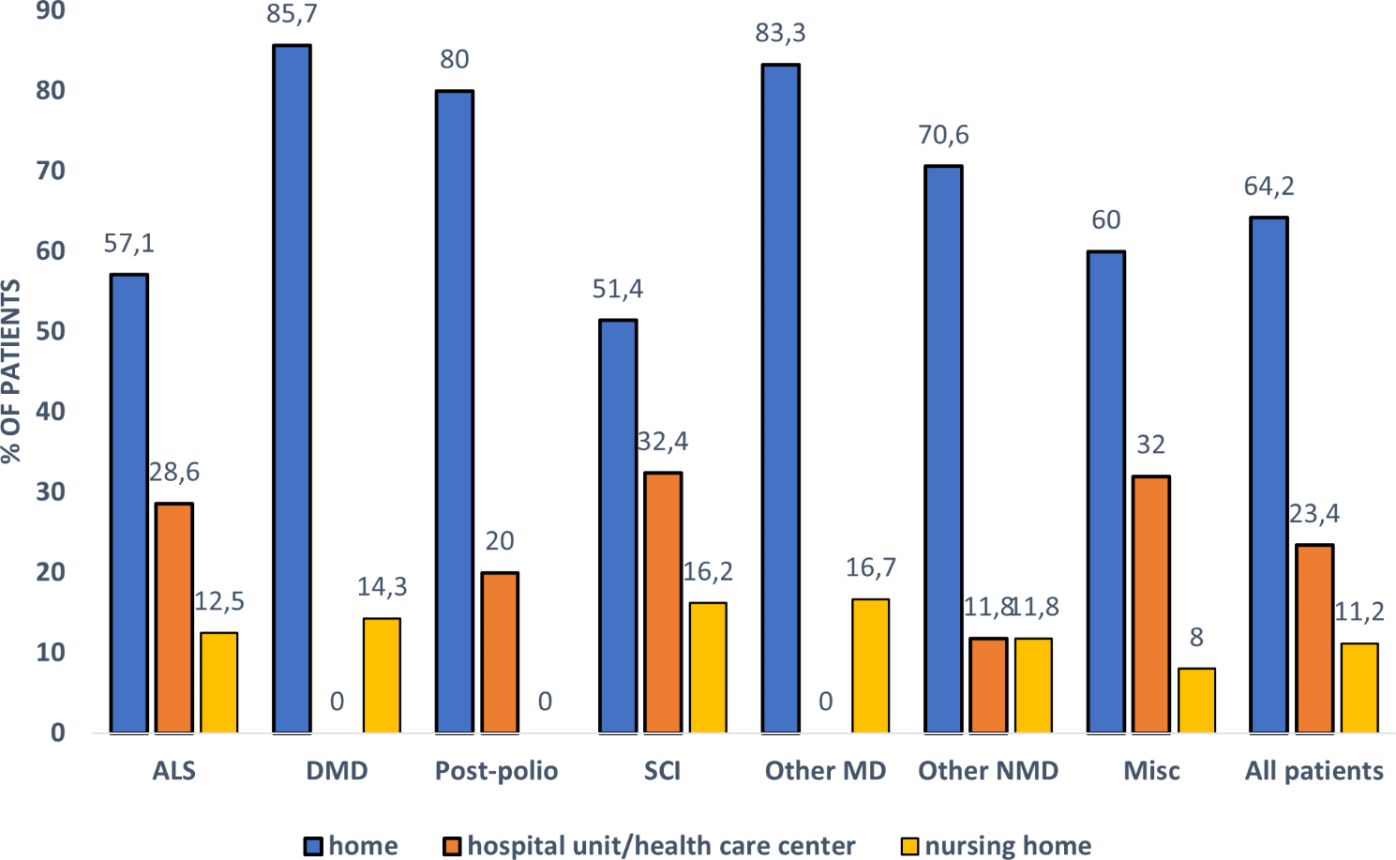
Distribution of patients (%) by place of residence divided in diagnostic groups. Abbreviations: ALS= amyotrophic lateral sclerosis, DMD= Duchenne muscular dystrophy, SCI= spinal cord diseases and injuries, MD=muscular dystrophies, NMD= neuromuscular diseases, Misc= miscellaneous

The data on diagnostic groups on HIMV is presented in Table 2. The duration of HIMV treatment for deceased patients at the time of death was 10.1 ± 10.5 years, and for those alive on December 31, 2022, it was 13.0 ± 12.4. Table 2 presents the durations of HIMV in different diagnostic groups—patients with post-polio syndrome, muscular dystrophies and other neuromuscular diseases had the longest treatment durations at death, and ALS patients had the shortest treatment durations at the time of death (Table 2). Figure 3 shows the number of deceased patients during follow-up period divided in groups by number of HIMV treatment years. Mortality rates were 3.4% at 1 year, 8.9% at 3 years, 16.2% at 5 years, and 29.6% at 10 years. Of all the HIMV treatments, 32% were elective. The percentages of elective initiations differ largely on the basis of the disease leading to HIMV (Fig. 4). Almost all the SCI patients began HIMV treatments in emergency situations, while over half of the HIMV treatments for DMD and ALS were elective. Most of the HIMV patients lived at home with 24-h caregiving (Fig. 5).

**Table 1:** Patient characteristics

*N*, (%) or hours ± SD	Number of patients *N* (%)	Sex (male) *N* (%)	New patients *N* (%)	HIMV usage h/day ± SD	Totally HIMV dependent* N (%)	Deceased patients during the follow-up *N* (%)
All patients	179 (100.0%)	123 (68.7%)	67 (37.4%)	22.5 ± 4.4	135** (82.8%)	84 (46.9%)
ALS	56 (31.3%)	34 (60.7%)	29 (51.8%)	23.5 ± 2.5	48 (96.0%)	30 (53.6%)
DMD	21 (11.7%)	21 (100.0%)	5 (23.8%)	24.0 ± 0.0	20 (100%)	11 (52.4%)
Post-polio	5 (2.8%)	2 (40,0%)	0	24.0 ± 0.0	4 (80.0%)	3 (60.0%)
SCI	37 (20.7%)	31 (83.8%)	16 (43.2%)	21.3 ± 6.5	25 (75.8%)	15 (40.5%)
Other MD	18 (10.1%)	12 (66.7%)	6 (33.3%)	22.5 ± 5.2	15 (83.3%)	8 (44.5%)
Other NMD	17 (9.5%)	7 (41.2%)	6 (35.3%)	21.7 ± 4.2	11 (73.3%)	6 (35.3%)
Miscellaneous	25 (14.0%)	16 (64.0%)	5 (20.0%)	20.5 ± 5.1	13 (54.5%)	11 (44.0%)

*usage 24 h/day

**information missing for 16 (8.9%) patients

DMD = Duchenne muscular dystrophy, SCI = spinal cord injuries and diseases, MD = muscular dystrophies, NMD = neuromuscular diseases

**Table 2:** Age of patients and durations of home invasive mechanical ventilation (HIMV) in different diagnostic groups

Years ± SD	All patients *N* = 179	ALS *N* = 56	DMD *N* = 21	Post-polio *N* = 5	SCI *N* = 37	Other MD *N* = 18	Other NMD *N* = 17	Miscellaneous *N* = 25
Age at diagnosis	36.06 ± 4.8	52.9 ± 10.6	7.1 ± 8.5	5.0 ± 4.8	42.9 ± 21.4	10.8 ± 15.7	26.8 ± 27.3	32.1 ± 27.9
Age at HIMV initiation	44.5 ± 20.9	57.0 ± 7.6	28.8 ± 8.1	15.6*	40.5 ± 22.0	16.9 ± 2.7	49.5 ± 22.1	36.5 ± 29.8
Age at death	60.3 ± 16.8	63.7 ± 14.4	41.9 ± 10.7	71.7 ± 6.9	61.5 ± 14.6	52.3 ± 20.4	70.0 ± 10.2	61.8 ± 21.3
Time from diagnosis to initiation of HIMV	7.2 ± 11.2	2.7 ± 3.2	20.9 ± 10.0	18.8 ± 26.8	1.6 ± 6.6	24.9 ± 14.1	7.9 ± 8.5	4.4 ± 8.4
Duration of HIMV for those alive on December 31, 2022	13.0 ± 12.4	4.3 ± 3.2	13.9 ± 8.6	60.0*	10.8 ± 10.0	24.7 ± 13.4	16.0 ± 16.0	18.1 ± 9.2
Duration of HIMV for the deceased	10.1 ± 10.5	7.7 ± 4.8	12.1 ± 10.2	42.8 ± 32.2	7.9 ± 6.0	13.6 ± 16.5	14.4 ± 13.6	7.0 ± 5.8

*Exact data known only for one patient

DMD = Duchenne muscular dystrophy, SCI = spinal cord injuries and diseases, MD = muscular dystrophies, NMD = neuromuscular diseases

## Discussion

The prevalence of HIMV in Finland has diminished during the eight-year follow-up from 2.4/100.000 on January 1,2015 to 1.8/100.000 on December 31,2022, respectively. The treatment periods were long, averaging over 10 years at the time of death, and varied largely depending on the underlying diagnosis. We observed no difference in treatment durations compared to our previous four-year follow-up study in 2019. This reaffirms the need for both patients and caregivers to anticipate a lengthy treatment period when planning HIMV treatment. Moreover, given the developing nature of NIV treatments and the possibility for patients to maintain their autonomy while using NIV, HIMV coupled with the high demands, and significant costs, it is advisable to prolong NIV treatments for as long as possible. Although the total number of HIMV patients is low (only 95 patients), the annual costs per patient are substantial — amounting to approximately €40 million.

Studies on HIMV prevalence are scarce. In recent European studies, the prevalence varies between 0.4 and 5.4/100.000 being lowest in Hungary and highest in Poland [5–7]. Our results are in line with these prevalence numbers. In Canada, the prevalence of ventilator-assisted individuals requiring long-term institutional care was 1.3/100,000 Canadians, but this number also includes NIV patients (30%) [8]. Most studies on HIMV are on ALS patients. The percentage of ALS patients using HIMV varies greatly. In Japan, 27% of ALS patients had undergone tracheostomy, an in Korea, the rate was 20,3% [9, 10]. European studies on ALS patients have revealed that 10.7% of ALS patients in Italy, 9.5% in Germany, and 4–7% in Norway and Sweden were tracheostomized and invasively ventilated [11–13].

Compared to our 2019 study, we defined the diagnostic groups more precisely in this study. This prevented us from making direct comparisons between the groups. Compared to our previous results, there seems to be a slight increase in the proportion of ALS (29% vs. 31%) and SCI patients (20% vs. 21%) and a decrease in the proportion of DMD (14% vs. 12%) [4]. In patients with advancing ALS who seek active treatment, HIMV becomes necessary when respiratory failure cannot be effectively managed by NIV or when severe bulbar symptoms prevent optimal NIV treatment. Nevertheless, the number of ALS patients who elect for e HIMV remains low in comparison to the overall ALS incidence in Finland. Annually approximately 250 new ALS diagnosis are made and four of these patients elect for HIMV treatment [14]. In comparison, approximately 2 out of 200 new SCI patients annually initiate HIMV [15]. Preventing all spinal cord injuries is not possible, and they may, in certain cases, lead to HIMV. In addition, recent study on SCIs reveals that the incidence and prevalence has slightly and steadily increased for both men and women from 1990 to 2019 [16]. According to our data, only a small number of our DMD patients are driven to HIMV, which is less than 0.5 patients per year (Table 1). According to our knowledge, this may reflect the evolution of NIV and the current guidelines that recommend NIV over HIMV for DMD patients [17, 18].

The treatment of patients suffering from chronic respiratory failure is evolving. While the prevalence of HIMV appears to be decreasing, NIV prevalence increases, particularly in the developed Western countries [7, 19, 20]. Many diseases that used to be treated with HIMV can now be treated with NIV — for example — muscular dystrophies and other neuromuscular diseases. In patients with ALS, long-term NIV has proved to provide survival benefit and improves QoL and well-being [21]. In DMD patients, the survival is equally good and long with NIV and HIMV [22]. However, safety and QoL is better with NIV—for example—verbal communication, sleeping, swallowing, comfort, and general acceptability are better [23].

Further, studies on HIMV treatment are scarce and even fewer have been conducted on the effects of treatments on QoL. Patients with chronic respiratory failure have a severe, uncurable, often end-stage disease, that markedly diminishes their functional ability and QoL. In earlier small studies on ALS patients, no difference has been found in QoL between patients treated with HIMV and NIV [24–26]. A recent large German study with 325 ALS patients has showed that being tracheostomized (n = 14) had a positive influence on QoL [27]. However, the number of tracheostomized patients in the study was small, and in patients whose respiratory insufficiency could not be corrected, the QoL was significantly reduced when compared to those who used NIV. Earlier, a Swiss workgroup has stated that tracheostomy and invasive mechanical ventilation appears to be associated with worse QoL and a higher risk of institutionalization than NIV [28]. In addition, a Cochrane review has noted that while tracheostomy and IMV prolong ALS patients’ life, they do not necessarily improve QoL when comparing it with NIV [21]. Since the results of studies on QoL of HIMV patients are conflicting, more research is needed to better understand the implications and impact of tracheostomy on QoL. It is also noteworthy that for ALS patients HIMV treatment increases the risk of a ‘locked-in’ state and causes a high burden for the caregivers [25, 28], Therefore, it is important to discuss these factors when considering the transitioning from NIV to HIMV.

Patients with chronic respiratory failure are at risk of an acute-on-chronic respiratory failure—for example— during airway infections. These situations can be life-threatening and lead to challenging decisions regarding treatment intensity. There are no studies that provide evidence-based practice guidelines for the use of non-invasive versus invasive mechanical ventilation (IMV) in acute life-threating situations [29]. Intubating and initiating IMV in these patients can result in ventilator dependency via tracheostomy and difficulties in weaning, thereby eventually leading to long-term HIMV. Advance care planning discussions should be initiated as early as possible to avoid undesired intubations and ensure that the treatment is performed in the manner that the patient desires [30]. In our study, elective HIMV initiations were most common in ALS and DMD patients 51.4% and 61.9%, respectively. Most HIMV patients prefer living at home. However, HIMV treatment demands advanced expertise and 24-h caregiving. For example, the patient’s physical condition or social circumstances may prevent them from living at home. Among our patients, DMD, other muscular dystrophy, and other NMD patients were most often living at home, whereas a larger proportion of SCI and ALS patients were living in the hospital, health care center units, or at nursing homes, thus reflecting the increased treatment needs of these patient groups.

HIMV treatment periods are lengthy, averaging over 10 years at the time of death. Treatment durations were the shortest in ALS patients and the longest in post-polio patients. The number of post-polio patients was small, only five patients, which lead to a situation in which the survival of one single patient has a significant effect on the results. It is noteworthy that of the patients who began treatment prior to 2015, 50 patients were still alive at the end of this study. Most of these patients had already transitioned to HIMV before the emergence of more advanced NIV techniques, such as mouth-piece ventilation (MPV). Today, patients with muscular dystrophy and other neuromuscular disease survive longer with NIV treatment modalities and can often avoid transition to HIMV altogether. In this study, over half of the patients with ALS and DMD had died. When compared ALS patients to the other diseases, the unfavorable prognosis in ALS coupled with the higher age leads to shorter treatment durations and a greater mortality rate. Most DMD patients in this study had transitioned in HIMV as young adults when NIV was not yet widely used; high mortality rates among these patients can be explained by the advanced age of the patients and the progressive nature of their disease.

### Limitations of the study

Our study has a few limitations. Finland lacks a register of both respiratory failure and HMV patients. Due to Finnish public healthcare system and legislation that guarantees equal rights to HIMV-dependent patients across Finland, we maintain, however, a registry of all HIMV-dependent patients in each hospital nationwide. The data was collected by personally contacting the physician responsible for HIMV patients in each hospital via telephone and email, and by sending a questionnaire to gather information on their HIMV patients. By personally contacting all hospitals, we ensured that all hospital districts were covered. We received responses from all hospital areas. However, it is still possible that a few patients are missing from our data. Since we could only collect data annually, it is possible that we missed patients who did not survive long enough to be included in this study. These would include patients whose treatment spanned over three months but had ended before the following January.

A longer follow-up period exceeding eight years would have revealed even more interesting data on the survival of HIMV patients. However, recent changes in Finnish legislation regarding patient registries have increased the complexity and cost associated with collecting national patient data [31] Unfortunately, this legislative alteration prevented us from continuing the follow-up of our patients. Additionally, it would have been valuable to have information on the number of patients who underwent NIV treatment before transitioning to HIMV, as well as the duration of their NIV therapy. Unfortunately, we do not have data on NIV use before HIMV, and obtaining this information is not feasible.

With regard to the results of the ALS patients, it should be noted that data on Finnish ALS patients might have only limited international generalizability. A recent study from Finland has demonstrated that there is a high frequency of the *C9orf72* and *SOD1* mutation variants in Finnish ALS patients [32]. ALS patients with the C9orf72 mutation had a significantly younger age at onset and a shorter survival time [32].

## Conclusion

HIMV is a rare treatment in Finland. The prevalence of HIMV treatment has decreased during the eight-year follow-up and was only 1.8/100,000 on December 31, 2022. Considering the small number of patients in this study, further studies with a longer follow-up period would be beneficial to confirm these results. NIV treatments have greatly evolved and has proven efficient in treating respiratory failure, prolonging life, and improving QoL. Given high demands, and the costs associated with HIMV, it is advisable to prolong NIV treatments for as long as possible.

## Data Availability

No datasets were generated or analysed during the current study.

## Abbreviations

ALS: amyotrophic lateral sclerosis
DMD: Duchenne muscular dystrophy
HIMV: home invasive mechanical ventilation
HMV: home mechanical ventilation
MC: muscular dystrophies
MPV: mouthpiece ventilation
NMD: neuromuscular diseases
NIV: non-invasive ventilation
SCI: spinal cord injuries, QoL: quality of life
